# A novel variant of the *IFITM5* gene within the 5′‐UTR causes neonatal transverse clavicular fracture: Expanding the genetic spectrum

**DOI:** 10.1002/mgg3.1287

**Published:** 2020-05-08

**Authors:** Dong Wu, Yuxin Wang, Huijuan Huang

**Affiliations:** ^1^ Department of Obstetrics and Gynecology 900 Hospital of the Joint Logistics Team or Dongfang Hospital Fuzhou Fujian People's Republic of China; ^2^ Department of Ultrasound 900 Hospital of the Joint Logistics Team or Dongfang Hospital Fuzhou Fujian People's Republic of China

**Keywords:** fracture, IFITM5, osteogenesis imperfecta, pathogenic variants, type V

## Abstract

**Background:**

Osteogenesis imperfecta (OI) type V is a rare heritable bone disorder caused by pathogenic variants of *IFITM5*. Only two mutated alleles in *IFITM5* have been identified worldwide, the role of which in OI pathology is not fully understood.

**Methods:**

A neonatal case of suspected OI, clinically manifested as a rare clavicle transection fracture with delayed early fracture healing, was studied. Subtle variants of OI‐associated genes were analyzed by whole exome sequencing and confirmed by Sanger sequencing.

**Results:**

A de novo heterozygous pathogenic variant of *IFITM5* within the 5′‐UTR (c.‐9C > A) was discovered in the proband. Bioinformatics analysis using a combination of various algorithms predicted that the variant would generate a new in‐frame start codon 9 bp upstream of the original and express a mutant IFITM5 protein with three additional amino acids (Met‐Glu‐Pro). After transfection into a eukaryocyte in vitro, the mutant IFITM5 construct produced a longer transcription product than that of wild‐type IFITM5.

**Conclusion:**

This study identified a novel pathogenic variant of *IFITM5*, which not only manifested the molecular characteristics of *IFITM5*, but also provided new evidence for the study of the molecular mechanisms of IFITM5 association with OI.

## Graphical abstract

A novel variant of the IFITM5 gene within the 5′‐UTR is identified. This study manifested the molecular characteristics of osteogenesis imperfecta. N‐terminal sequences are important to the function of IFITM5.

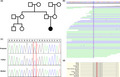

## 1 INTRODUCTION

Osteogenesis imperfecta (OI) describes a group of genetic disorders characterized principally by bone fragility and low bone mass. OI patients are more susceptible to fracture, often after minimal trauma or with no apparent cause. OI patients may also suffer from dentinogenesis imperfecta, hearing loss, muscle weakness, loose joints, and/or blue sclera. OI was initially classified as four different types according to a scheme developed by David Sillence based on family history, clinical presentation and radiological findings (Sillence, Senn, & Danks, 1979). OI Type I (OI1) is the mildest while type II (OI2) is the most severe. Approximately 90% of all cases with clinically confirmed OI are autosomal dominant with pathogenic variants in one of the two genes that code for type I collagen alpha chains, *COL1A1* (MIM + 120,150) and *COL1A2* (MIM *120,160) (Lin et al., 2019; Rauch & Glorieux, 2004; Wenstrup, Willing, Starman, & Byers, 1990). Genetic changes in *COL1A1* and *COL1A2* cause COL1A1/2‐related OIs by reducing the quantity of type I collagen molecules secreted or altering their structure.

Because genetic factors are increasingly used to define different types of OI (MIM #166200, #166210, #259420, #166220, #610967, #613982, #610682, #610915, #259440, #613848, #610968, #613849, #614856, #615066, #615220, #616229, #616507, #617952, #301014, and #618644 for types I–XX of the disease), at least 18 genes, including *COLIA1* and *COLIA2*, have been identified as possibly giving rise to an OI phenotype. Osteogenesis imperfecta type V (OI5) is an autosomal dominant form of OI, first described by Glorieux in 2000 (Glorieux et al., 2000). The disorder consists of ~4%–5% of OI cases (Rauch & Glorieux, 2004), having distinctive clinical and histologic characteristics, including calcification of the forearm interosseous membrane, anterior dislocation of the radial head, a subphyseal metaphyseal radiodense line, and hyperplastic callus formation. In 2012, two research groups identified the interferon induced transmembrane protein 5 (*IFITM5*) gene (MIM *614,757), which encodes bone restricted IFITM‐like protein (BRIL), as responsible for causing OI5 (Cho et al., 2012; Semler et al., 2012).

In light of present knowledge, only three *IFITM5* pathogenic variants have been identified that are associated with OI: a recurrent C‐T transition (c.‐14C > T) in the 5′‐UTR and two missense variants in codon 40 (serine) in exon 1 (Farber et al., 2014; Hanagata, 2016; Lim et al., 2019). The c.‐14C > T variant, the most frequently observed in OI5, introduces a new upstream in‐frame start codon, which results in the mutant IFITM5 protein having five additional amino acids in the N terminus (MALEP‐IFITM5). As with all other types of OI, patients with OI5 demonstrate high phenotypic variability and may suffer from a highly variable degree of bone fragility, even when carrying the same variant. The missense IFITM5 variants (p.Ser40Leu; p.Ser40Trp) cause a more severe OI phenotype that is similar to OI6, but not OI5, such as blue sclera and dentinogenesis imperfect (Fitzgerald et al., 2013; Grover et al., 2013; Rauch et al., 2013). In the present study, we found a novel heterozygous pathogenic variant in the 5′‐UTR of *IFITM5*, occurring de novo and resulting in a transverse right mid‐clavicular fracture of a Chinese newborn infant.

## 2 MATERIALS AND METHODS

### 2.1 Ethical compliance

The protocols for this study were evaluated and approved by the Ethics Committee of 900 Hospital of the Joint Logistics Team (formerly known as Fuzhou General Hospital). Written informed consent for genetic analyses of samples and for publication of test results and clinical data were obtained from the parents of the proband.

### 2.2 Information about the patient

**FIGURE 1:**
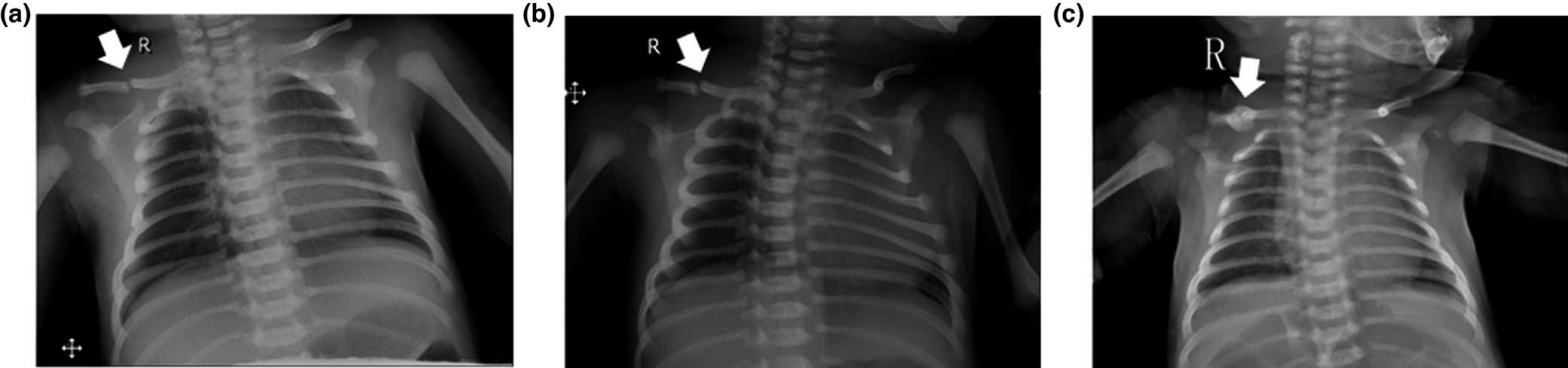
Radiographic features of the patient. Chest X‐rays at (a) 2 days of age; (b) 9 days of age; and (c) 30 days of age

The proband was a newborn female with the novel form of OI. She was the first child of nonconsanguineous Chinese parents, born by spontaneous vaginal delivery at 40 weeks of gestation (APGAR 3‐9‐10). At birth, she presented with a weight of 3,350 g, length of 49.5 cm, and head circumference of 34.5 cm, all within the normal range (−2 to +2 *SD*). The patient was referred to the neonatal department because of excessive crying and irritability on postnatal day 2. Physical examination revealed her right Moro reflex was absent and the muscle tone of her right arm slightly deficient. The motion, muscle tone, and tendon reflexes of other limbs were all within the normal range. There were no apparent skeletal abnormalities or unusual facial features. A transverse right mid‐clavicular fracture was observed in a chest X‐ray (Figure 1a). Given the uncommon type of fracture in the absence of any apparent history of trauma in the perinatal and postnatal periods, the neonate was suspected of having a form of congenital OI. Blood biochemical analysis, including calcium, phosphorus, alkaline phosphatase, and parathyroid hormone (PTH), were normal, as was her hearing assessment and ophthalmological examination. There was no family history of OI or other skeletal diseases. Re‐examination via X‐ray revealed that there was no apparent callus formation 9 days after birth, although it had formed completely 30 days later (Figure 1 and c), suggesting delayed early healing of the fracture.

### 2.3 Exome sequencing

To identify the pathogenic variant, whole‐exome next‐generation sequencing (NGS) was conducted using an Illumina Hiseq X™ platform (Illumina) with PE150 (paired‐end sequencing, 150 bp reads). Briefly, all exomes were captured using Agilent's SureSelect Human All Exon V6 exome library (Agilent Technologies). Reads were aligned using Novoalign V3.09.00 software (Novocraft Technologies) to the human reference genome GRCh37/hg19. SNPs and short indels were called using SAMtools v1.3.1 (Li et al., 2009) and VarScan software v2.3.9 (Koboldt et al., 2009). Variants were annotated using Alamut‐Batch standalone v1.9 software (Interactive Biosoftware, France). Protein‐impacting variants that were rare (minor allele frequency < 10%) or novel were preferentially explored. A phenotype‐driven analysis of the submitted specimen was undertaken, focusing on 36 genes associated with OI and decreased bone density, including *ALPL* (MIM *171,760), *ANO5* (MIM *608,662), *B4GALT7* (MIM *604,327), *BMP1* (MIM *112,264), *CLCN5* (MIM *300,008), *COL1A1* (MIM + 120,150), *COL1A2* (MIM *120,160), *CREB3L1* (MIM *616,215),*CRTAP* (MIM *605,497), *DMP1* (MIM *600,980), *ENPP1* (MIM *173,335), *FGF23* (MIM *605,380), *FKBP10* (MIM *607,063), *GNAS* (MIM *139,320), *GORAB* (MIM *607,983), *IFITM5* (MIM *614,757), *LMNA* (MIM *150,330), *LRP5* (MIM *603,506), *MBTPS2* (MIM *300,294), *MAFB* (MIM *608,968), *MESD* (MIM *607,783), *MMP2* (MIM *120,360), *NOTCH2* (MIM *600,275), *P3H1* (MIM *610,339), *PHEX* (MIM *300,550), *PLOD2* (MIM *601,865), *PPIB* (MIM *123,841), *SERPINF1* (MIM *172,860), *SERPINH1* (MIM *600,943), *SLC34A3* (MIM *609,826), *SP7* (MIM *606,633), *SPARC* (MIM *182,120), *TENT5A* (MIM *611,357), *TMEM38B* (MIM *611,236), *TNFRSF11A* (MIM *603,499), and *WNT1* (MIM *164,820). For this specimen, 99.25% of targeted exons and splicing junctions of genes listed were covered at a sequencing depth no less than 10×, with 98.83% covered at a sequencing depth no less than 20×. Variants were curated manually using the American College of Medical Genetics (ACMG) scoring system (Nykamp et al., 2017; Richards et al., 2015). All genes listed above were evaluated for large deletions and/or duplications. All candidate variants detected by NGS in addition to those with total quality scores of less than 500 in the candidate genes were confirmed using Sanger sequencing.

### 2.4 Sanger sequencing

Amplification and Sanger sequencing of the *IFITM5* were performed as described previously (Cho et al., 2012; Semler et al., 2012). Amplicons were generated using 10 ng of genomic DNA using Ex Taq polymerase (Takara, China). PCR conditions for this amplification were as follows: 95°C for 5 min, 30× (95°C for 30 s, 60°C for 45 s, 72°C for 30 s), then 72°C for 5 min. Primers used were: forward: 5′‐CTCCCAGATGGGATGTCTGTCAG‐3′ and reverse: 5′‐TTCCCGGCCATACTGATAGCTGTG‐3′. Products were verified by agarose gel electrophoresis then sequenced using Sanger sequencing using the same primers on a 3730 genetic analyzer (Applied Biosystems). PCR products of normal controls (100 alleles) were analyzed by simultaneous direct sequencing so as to exclude the possibility of polymorphism.

### 2.5 Bioinformatics analysis

Bioinformatics prediction analyses were performed using AGVGD, DANN, DEOGEN2, EIGEN, FATHMM, LRT, MAPP, M‐CAP, METALR, METASVM, VARIANTASSESSOR, VARIANTTASTER, MVP, PRIMATEAI, PROVEAN, REVEL, and SIFT. All algorithms were integrated into Alamut® Visual v2.11 software (Interactive Biosoftware, France) or at the Varsome website (http://varsome.com). Prediction of translation initiation sites was performed by DNA TIS Miner (http://dnafsminer.bic.nus.edu.sg/) and ATGpr (http://atgpr.dbcls.jp/).

### 2.6 Site‐directed mutagenesis of *IFITM5*

Wild‐type *IFITM5* cDNA was amplified from the cDNA of human primary osteogenic sarcoma Saos‐2 cells using forward (5′‐ACCAGTCTGAGTGTGGAAGAGAC‐3′) and reverse (5′‐CTGGAACCAGGCACTTTTAATCG‐3′) primer sequences. Two mutated *IFITM5* cDNA (c.‐9C > A or in cis with c.2T > C) were generated by site‐directed mutagenesis using primers designed in accordance with the instructions for site‐directed mutagenesis reagents (QuikChange site‐directed mutagenesis kit, Stratagene) (Bachman, 2013). Wild‐type and mutant *IFITM5* were cloned into a pcDNA3.1 vector (Thermo Fisher Scientific) with HindIII and EcoRI (Takara). All recombinant vectors were confirmed by colony PCR and DNA sequencing.

### 2.7 Transfection and Western blot analysis

HEK293T cells were cultured in Dulbecco's Modified Eagle Media (DMEM) supplemented with 10% fetal bovine serum (FBS) and antibiotics (all from Invitrogen). The cells were transfected with recombinant vectors expressing wild‐type or mutant IFITM5 using Lipofectamine 2000 reagent (Invitrogen), in accordance with the manufacturer's recommendations. After transfection and culture for 48 hr, the HEK293T cells were harvested and lysed. Western blotting was performed on crude cell homogenates using an anti‐IFITM5 polyclonal antibody.

## 3 RESULTS

### 3.1 Subtle variants detection

**FIGURE 2:**
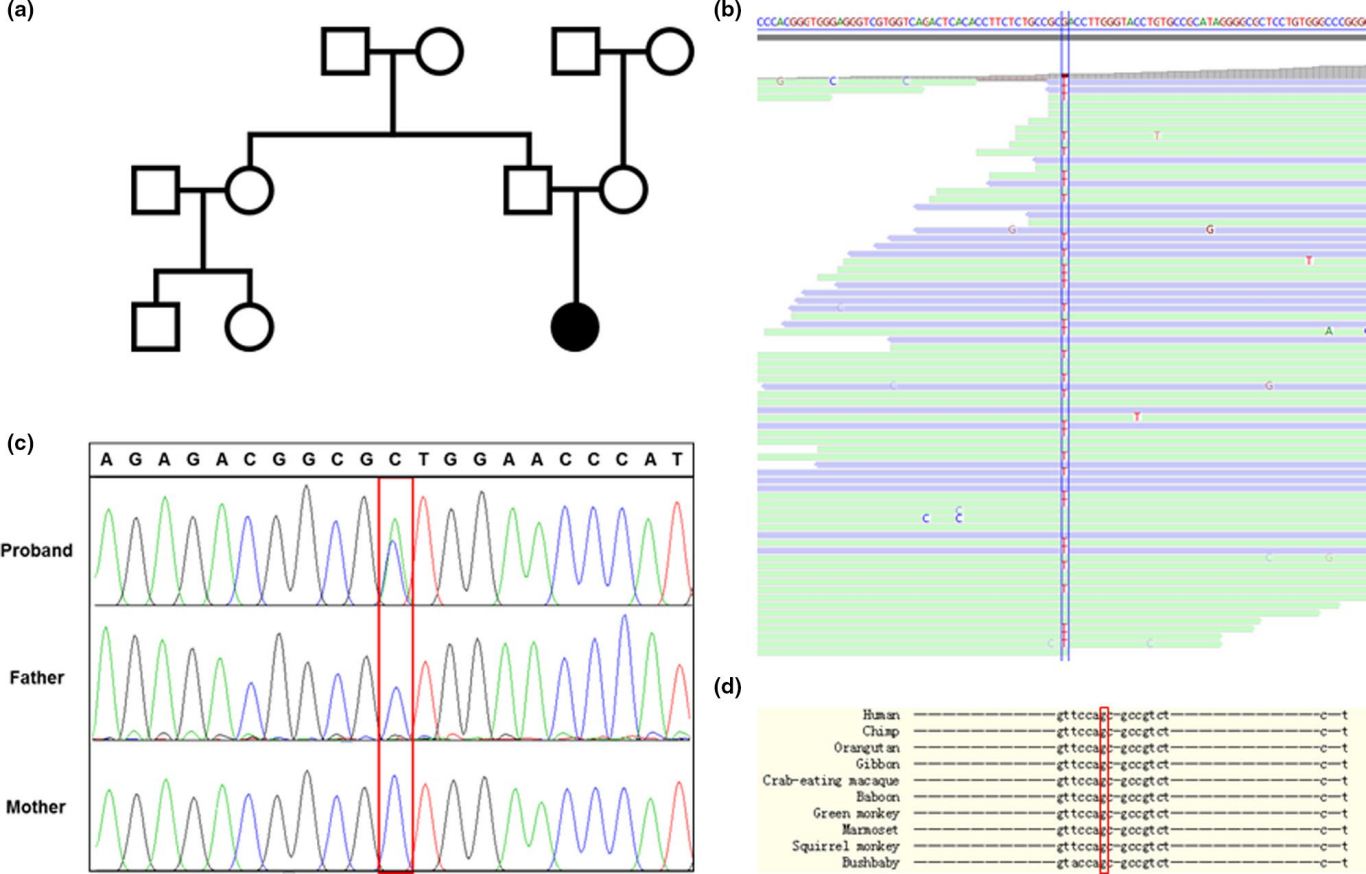
(a) Pedigree. (b) Alignment of the next‐generation sequencing reads as viewed in Alamut software. The letter T represents the 5′‐UTR variant in *IFITM5*. (c) Sanger sequencing results for the *IFITM5* variant identified by next‐generation sequencing. (d) Multiple nucleoside sequence alignment demonstrates evolutionary conservation of the G allele where the variant was identified

A heterozygous variant within the 5′‐UTR of *IFITM5* was identified in the genomic DNA of the proband by NGS. As shown in Figure 2, of 74 unique reads at the genomic position chr11:299499 (GRCh37/hg19), where the reference allele was G, 32 displayed a heterozygous variant T (Chr11:g.299499G > T). The variant was in an intronic position located 9 bp upstream of the transcription start site (NM_001025295.2:c.‐9C > A). Segregation analysis by Sanger sequencing indicated that the variant was not detected in the genomes of either parent (Figure 2a and c), indicating that this was a de novo variant. This variant was also not found after searching the Broad database (GnomAD) with good coverage (31.9×). Analysis of DNA sequence conservation indicated that the position of this allele (G) is conserved in 48 mammals, although the (C) allele is observed in some nonmammalian vertebrate species, indicating that this particular substitution might not be tolerated (Figure 2d).

### 3.2 Bioinformatics prediction

**FIGURE 3:**
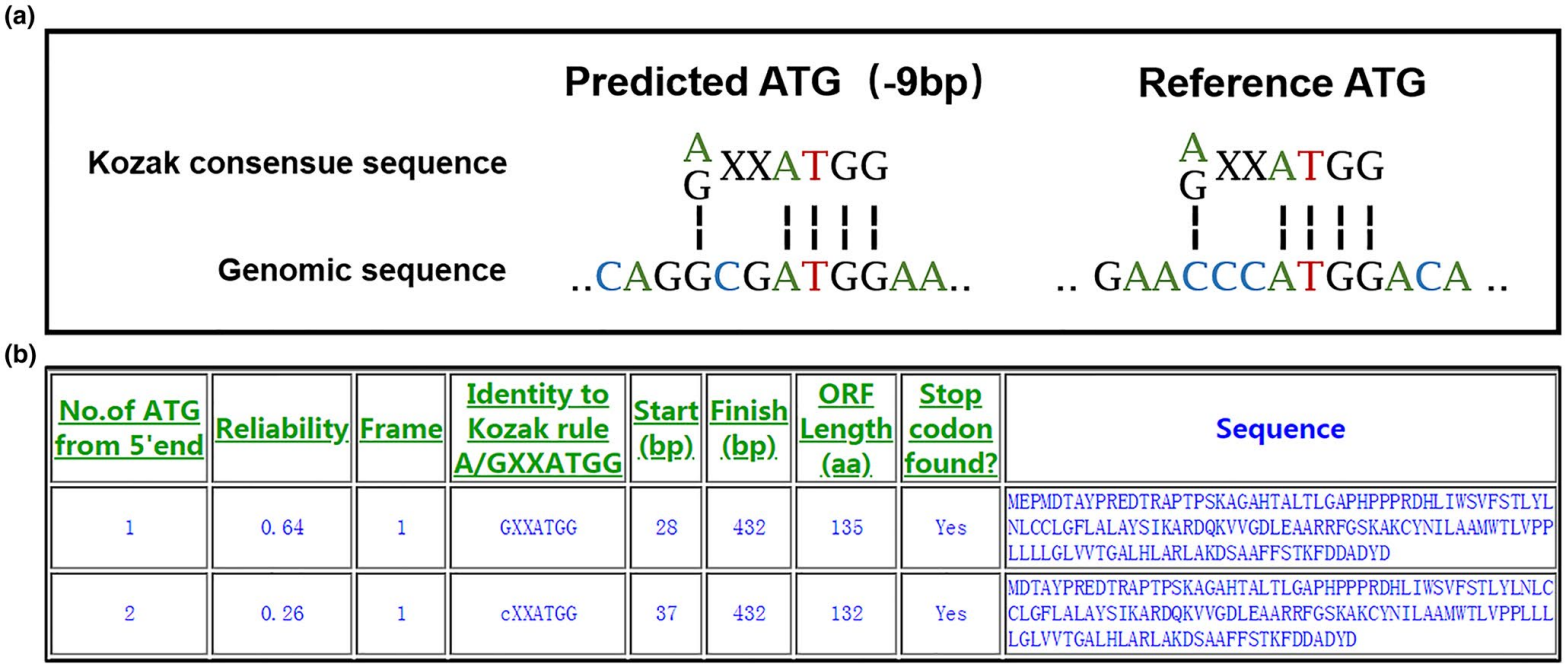
(a) Comparison of the Kozak sequence (A/G) XXATGG with the reference and variant alleles of *IFITM5*. (b) Translation initiation site prediction of the mutant *IFITM5* in DNA TIS Miner. No. 1 ATP is introduced by the variant c.‐9C > A; No.2 ATP is the original start codon

DANN prediction software, queried from within Varsome, suggested that this variant may have a deleterious effect on *IFITM5* or its gene product (scores = 0.9341). However, all 16 other programs (AGVGD, DEOGEN2, EIGEN, FATHMM, LRT, MAPP, M‐CAP, METALR, METASVM, MUTATIONASSESSOR, MUTATIONTASTER, MVP, PRIMATEAI, PROVEAN, REVEL, and SIFT) failed to provide available predictive results. Because a different change (c.‐14C > T) nearby has already been determined to be pathogenic, in which an in‐frame translation of the upstream start codon had occurred with the addition of five amino acids (MALEP) at the N terminus of IFITM5 (MALEP‐IFITM5), protein initiation translation analysis was performed on the mutant *IFITM5* DNA using DNA TIS Miner and ATGpr. Both algorithms predicted that the *IFITM5* variant was embedded in a Kozak sequence and that the new start codon (AUG) was stronger than the original. When the upstream AUG was utilized, three amino acids (Met‐Glu‐Pro, MEP) were added to the N terminus of the IFITM5 protein (MEP‐IFITM5), thereby increasing its size from 132 to 135 amino acids (Figure 3).

### 3.3 Immunoblot analysis of mutant *IFITM5* products

**FIGURE 4:**
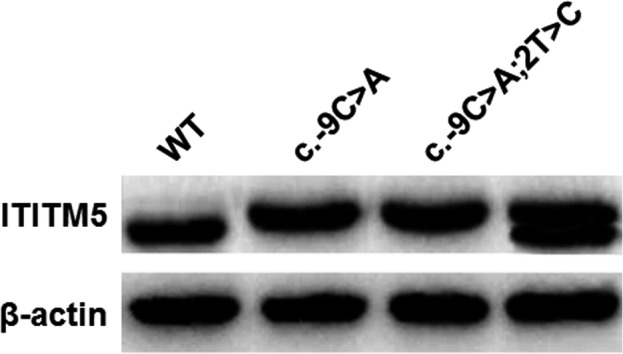
In vitro translation assay for the c.‐9C > A variant. HEK293T cells were transiently transfected with constructs containing wild‐type (WT) or mutant IFITM5. The translation product of the mutant IFITM5 was slightly larger than that of the wild‐type. Co‐transfection with wild‐type and mutant constructs produced double bands. Beta‐actin was used as an internal control

To test the potential for the mutated 5′‐UTR sequence to be recognized as the start site for translation, we examined whether the mutant *IFITM5* construct (c.‐9C > A) could be expressed alone or coexpressed with the wild‐type version in eukaryotic cells. Immunoblot analysis of transfected cell lysates revealed that the mutant and the wild‐type constructs were translated separately or simultaneously in transfected 293T cells with the translation products of the mutant being slightly larger, confirming that the expression of the mutant constructs initiated at the start codon generated by the variant rather than at the original start codon (Figure 4).

## 4 DISCUSSION

This study identifies a de novo heterozygous pathogenic variant of *IFITM5* (NM_001025295.2:c.‐9C > A) carried by a newborn with a fracture in her right clavicle. According to ACMG guidelines with minor adjustments, the *IFITM5* c.‐9C > A variant was classified as likely pathogenic (Criteria: PS2‐Moderate, PS3‐Moderate, PM2 and PP3). This is the third variant allele of *IFITM5* that may be associated with OI found so far (Hanagata, 2016). Similar to the recurrent c.‐14C > T variant, this variant was located in the evolutionarily conserved allele of the 5'‐UTR of *IFITM5* resulting in a mutated IFITM5 protein with additional amino acids (MEP‐IFITM5). In terms of interpretation of the variant, the priority of candidate variants (levels 1–9) was evaluated based on the possibility of pathogenicity. Initially, the variant received the lowest priority (level 9) due to its location in the noncoding region without positive bioinformatics analysis evidence (based on three prediction tools) and the low population frequency. Our attention was aroused when the bioinformatics analysis suggested that the variant was located within 15 bp of a known pathogenic variant. The value of bioinformatics analysis in the interpretation of genetic variation is becoming increasingly recognized. Various bioinformatics analyses based on phenotype driven, protein function prediction, priority evaluation, and other strategies are considered to be effective in improving the efficiency and accuracy of variant interpretation (Maver et al., 2016; Jiang et al., 2019; Roy et al., 2018). In the present study, 17 bioinformatics prediction tools from two platforms were adopted to finally obtain one positive prediction (DANN) (Quang, Chen, & Xie, 2015). Furthermore, after obtaining the results of functional testing, the variant was successfully classified. This indicates that for different variants, especially those in the noncoding region, a suitable bioinformatics tool should be selected based on the type of variant so as to achieve valuable prediction and analysis results.

Although ununited fractures are not rare in OIs (prevalence 15%–20%) (Agarwal & Joseph, 2005), the current studies of *IFITM5*‐associated OIs are principally focused on the presence of hyperplastic callus formation, but seldom consider other aspects of fracture healing. Except for fractures with no apparent cause and mild delayed early healing of fractures, the proband had no visible symptoms of OI5 such as hyperplastic callus formation, calcification of the forearm interosseous membrane, or radial head dislocation. This is the first report of a delay in fracture healing. This demonstrates that OI caused by an *IFITM5* variant can be highly clinically heterogeneous. According to previous studies, typical features may occur in children with OI5 from months to years after birth (Fitzgerald et al., 2013; Guillen‐Navarro et al., 2014; Shapiro et al., 2013). Considering that the proband was a newborn, the absence of typical clinical manifestations may be related to being young. However, as the child will receive frequent follow‐up visits and treatment in the future, we may not be able to accurately determine whether the child will present characteristics typical of OI5, such as hyperplastic callus formation, other OI symptoms, or even no apparent symptoms of OI. Therefore, we recommend that when patients are diagnosed with suspected OI, they should be tested with high‐throughput multigene analysis, including that of the *IFITM5* in order to obtain earlier diagnosis and treatment opportunities, no matter whether typical clinical manifestations have occurred or not.

The difference between the recurrent MALEP‐IFITM5 protein and the MEP‐IFITM5 protein identified in this study is the presence of only two additional amino acids, so we can easily deduce that these two mutant IFITM5 proteins exhibit the same or similar roles in OI pathology. If three or five amino acids added at the N terminus of IFITM5 are pathogenic, it is unknown whether the addition of one, two, or other numbers of amino acids would be pathogenic. It is possible that there are modification sites and protein interaction sites at the N terminus of IFITM5 critical to its function. However, although the recurrent c.‐14C > T variant is found in almost all patients with OI5, the mechanism by which it causes OI remains largely unexplored. According to bioinformatic predictions (a gnomAD pLI score of 0 and a DECIPHER HI index of 56.57%), *IFITM5* is likely to be loss‐of‐function and haploinsufficiency tolerant (Hanagata et al., 2011). Although the role of IFITM5 in bone mineralization has been confirmed (Moffatt et al., 2008), we cannot come to a definite conclusion about the effects of the c.‐14C > T variant from the results of previous studies, because the results of whether the c.‐14C > T variant leads to reduced or increased mineralization are conflicting (Blouin et al., 2017; Lietman et al., 2015; Rauch et al., 2018). Until now, it has been universally accepted that the c.‐14C > T variant acts in a neomorphic fashion. The mechanism might be associated with upregulation of pigment epithelium‐derived factor (PEDF), induction of an inflammatory response or other effects (Farber et al., 2014; Rauch et al., 2018), warranting further research. For the *IFITM5* c.‐9C > A variant identified in this study, although we have established it can produce a long form IFITM5 protein, its genuine role in pathogenesis remains to be further investigated in cell or animal models, especially considering that bone samples from such patients are limited, as surgical procedures may produce a number of deleterious effects, such as increased risk of hyperplastic callus (Cheung, Glorieux, & Rauch, 2007). In any case, the discovery of this novel *IFITM5* variant suggests that additional studies should focus on the N‐terminal function of IFITM5 when investigating the molecular mechanisms of OI associated with *IFITM5*.

## CONFLICT OF INTEREST

All authors state that they have no conflicts of interest.

## AUTHOR CONTRIBUTIONS

Dong Wu and Huijuan Huang designed the research; Dong Wu performed research; Yuxin Wang performed prenatal and postnatal imaging analysis.

## Data Availability

Some or all data, models, or code generated or used during the study are available in a repository or online in accordance with funder data retention policies.
